# Primary TRAF7-Mutated Myxoid Mesenchymal Tumor With Predominant Epithelioid Morphology: Expanding the Morphologic Spectrum of TRAF7-Mutated Myxoid Mesenchymal Tumors

**DOI:** 10.7759/cureus.100187

**Published:** 2025-12-27

**Authors:** Rana Naous, Joel Mayerson

**Affiliations:** 1 Pathology, The Ohio State University Wexner Medical Center, Columbus, USA; 2 Orthopedics, The Ohio State University Wexner Medical Center, Columbus, USA

**Keywords:** epithelioid, mesenchymal, myxoid, spindle cell, traf7

## Abstract

*TRAF7*-mutated mesenchymal tumors are an emerging entity with very few cases reported in the literature so far. All *TRAF7*-mutated myxoid mesenchymal tumors reported to date have harbored a low-grade spindle cell morphology as the primary presentation and involved pediatric and adult age groups with variable clinical courses. Given its rare nature, the histomorphologic spectrum and clinical behavior of TRAF7-mutated myxoid mesenchymal tumors are difficult to determine and are best regarded as uncertain. Herein, we report the first known case of TRAF7-mutated myxoid mesenchymal tumor with predominant epithelioid morphology as the primary presentation involving the left thigh of an adult female patient, thus expanding the morphologic spectrum of TRAF7-mutated myxoid mesenchymal tumors.

## Introduction

*TRAF7* (TNF receptor-associated factor 7) is a member of the tumor necrosis factor receptor-associated factors family, which act as signal transducers for members of the TNF receptor superfamily [1]. In soft tissue tumors, frequent *TRAF7* gene mutations have been associated mainly with intraneural perineuriomas [2]. Recently, *TRAF7*-mutated mesenchymal tumors, with missense mutations involving the C-terminal WD40 domains of *TRAF7* gene, have been reported as an emerging entity whereby only four cases thus far have been documented in the literature with variable clinical courses [3,4]. All *TRAF7*-mutated myxoid mesenchymal tumors reported to date have harbored a low-grade spindle cell morphology as the primary presentation. The initial report on such tumors occurred in adults and presented as deep soft tissue tumors, involving trunk and extremities, with an aggressive clinical course [3]. On the other hand, a recent pediatric case involving the bowel wall showed a biphasic morphology with low-grade spindle cell areas adjacent to hypercellular areas with significant cytologic atypia and increased mitotic activity and was reported to harbor a potentially indolent behavior thus far [4]. Given their rare nature, the histomorphologic spectrum and clinical behavior of *TRAF7*-mutated myxoid mesenchymal tumors is difficult to determine. Epithelioid morphology as the primary presentation in such tumors has not been described as of yet. Herein, we report the first documented case, to our knowledge, of *TRAF7*-mutated myxoid mesenchymal tumor with predominant epithelioid morphology as the primary presentation involving the left thigh of an adult female patient. As myxoid soft-tissue tumors, including myxofibrosarcomas, low-grade fibro-myxoid sarcomas, myxoid liposarcomas, myoepithelial tumors and myxoinflammatory fibroblastic sarcomas, among others, are inherently diagnostically challenging given their morphologic overlap, in this case report, we expand the histopathologic spectrum of such rare and diagnostically challenging myxoid mesenchymal tumor, discuss pitfalls inherent to the differential diagnosis and review the literature on this unique entity.

## Case presentation

**Figure 1:**
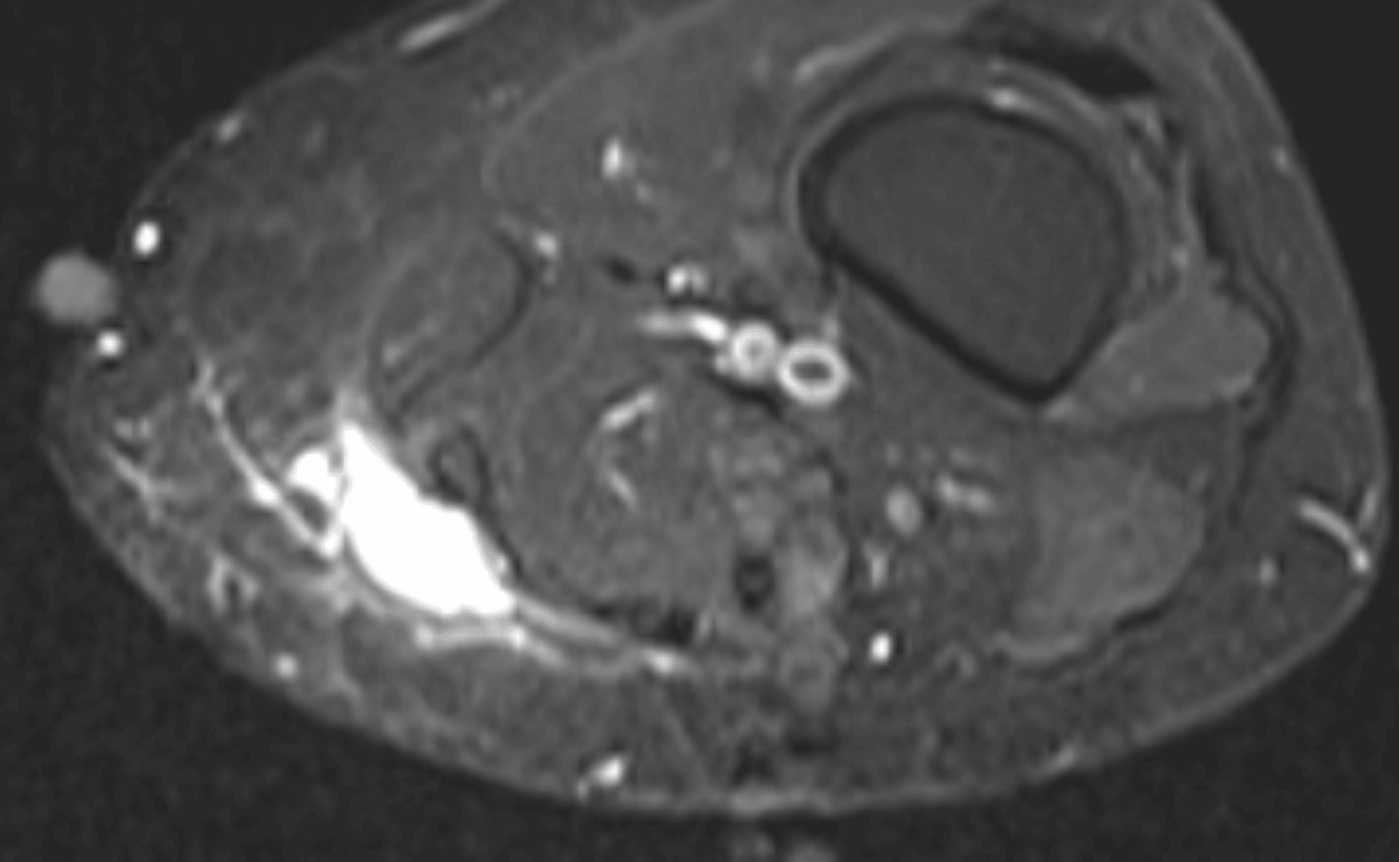
MRI of left thigh mass MRI image showing an ovoid mass along the surface of the superficial fascia abutting the posteromedial aspect of the gracilis and sartorius muscles.

A 54-year-old female non-smoker with past medical history of diabetes and mixed hyperlipidemia presented with a left distal thigh palpable mass that she had discovered one month prior during a shower. On clinical examination, the mass was firm, deep, semimobile and non-tender. The patient had no history of trauma or cancer. Radiologic magnetic resonance imaging (MRI) identified a 3.0x2.3x1.5 cm well-circumscribed ovoid mass along the surface of the superficial fascia abutting the posteromedial aspect of the gracilis and sartorius muscles (Figure 1). The mass was isointense to muscle on T1 images and showed an intermediate but heterogeneous signal intensity on STIR images, with thick peripheral rim enhancement and low signal intensity rim following contrast administration. 

**Figure 2:**
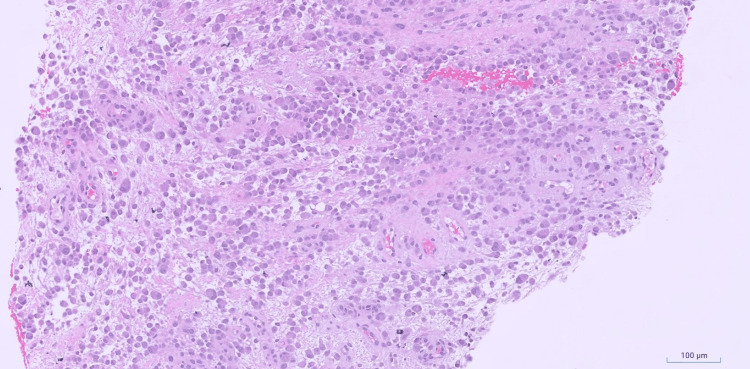
TRAF7-mutated myxoid mesenchymal tumor with epithelioid features - intermediate power Intermediate power magnification demonstrating loose sheet-like proliferation of epithelioid tumor cells with moderate atypia in a myxoid background (hematoxylin and eosin (H&E), 10x).

**Figure 3:**
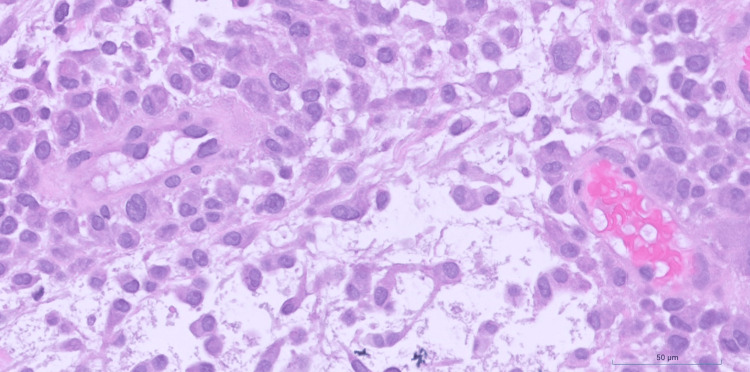
TRAF7-mutated myxoid mesenchymal tumor with epithelioid features - high power High power magnification showing the epithelioid nature of the tumor cells with plump cytoplasm, round to ovoid nuclei, prominent nucleoli and moderate atypia (hematoxylin and eosin (H&E), 40x).

A biopsy was performed, and microscopic examination revealed a discohesive or loose sheet-like proliferation of epithelioid cells with plump cytoplasm, round to ovoid nuclei with prominent nucleoli, occasional bi- and multinucleation and moderate atypia (Figures 2, 3).

**Figure 4:**
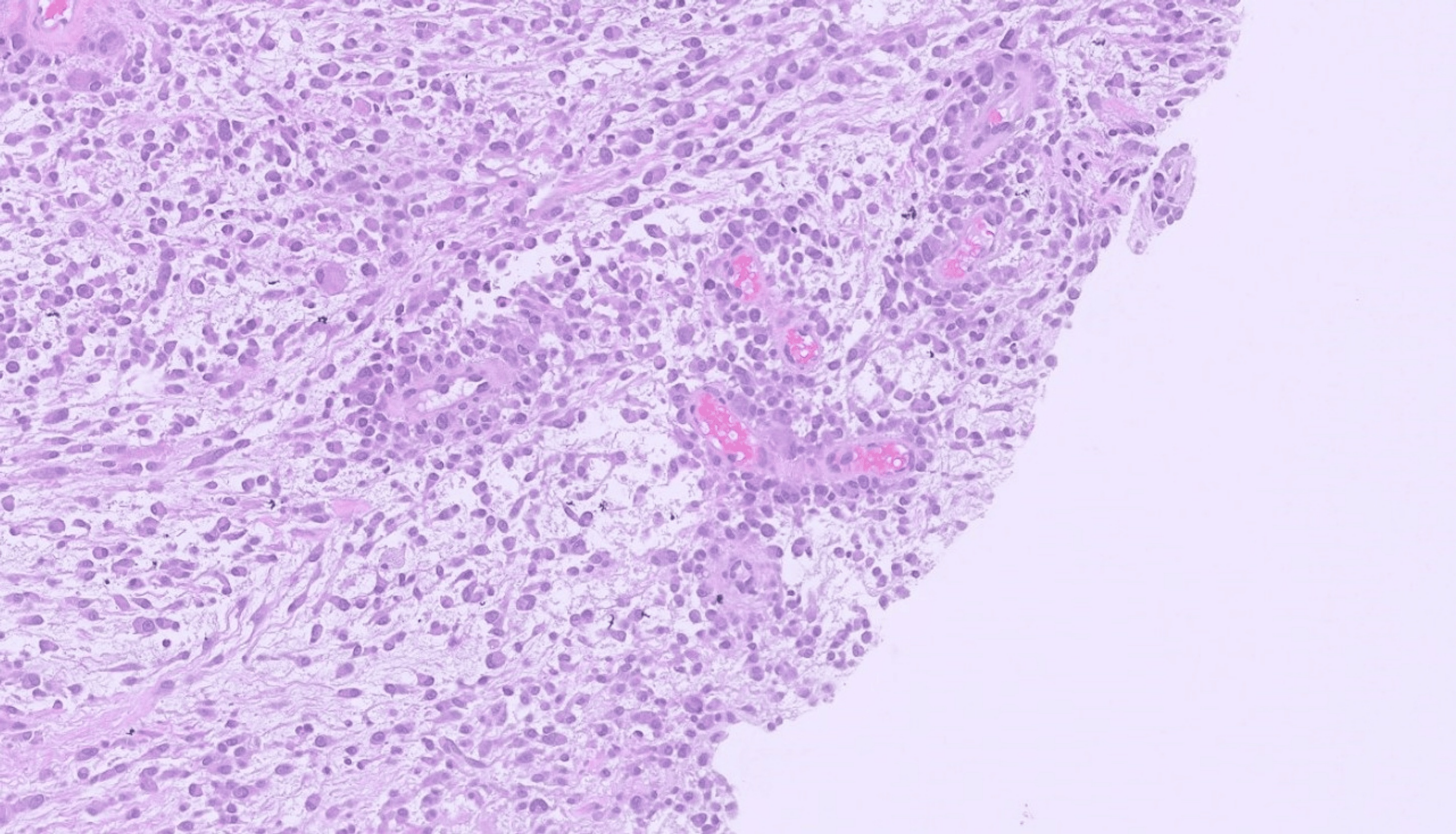
TRAF7-mutated myxoid mesenchymal tumor with epithelioid features - perivascular condensation The tumor showing perivascular condensation and variable perivascular hyalinization (hematoxylin and eosin (H&E), 10x).

The tumor cells are set in a myxoid to occasionally hyalinized stroma with variable perivascular hyalinization and occasional perivascular condensation (Figure 4).

**Figure 5:**
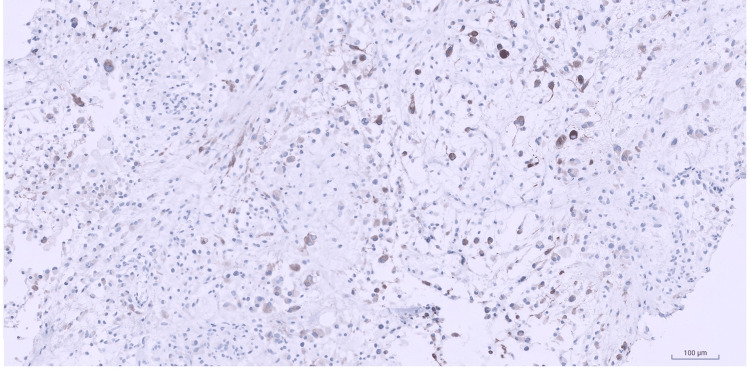
Desmin immunostain Desmin immunostain highlighting the tumor cells in a focal patchy non-specific manner (Immunohistochemistry (IHC), 10x).

Mitotic figures were rare and there was no definite evidence of necrosis. Immunohistochemical stains were performed and showed focal patchy non-specific staining for desmin (Figure 5).

**Figure 6:**
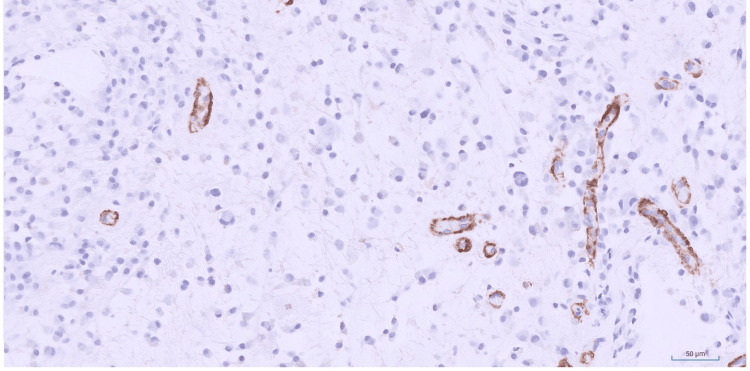
SMA immunostain SMA immunostain highlighting background vessels with negative staining in the tumor cells (Immunohistochemistry (IHC), 20x).

Myoid markers including SMA (Figure 6), smooth muscle myosin heavy chain (SMMS), myogenin, and H-caldesmon were negative.

**Figure 7:**
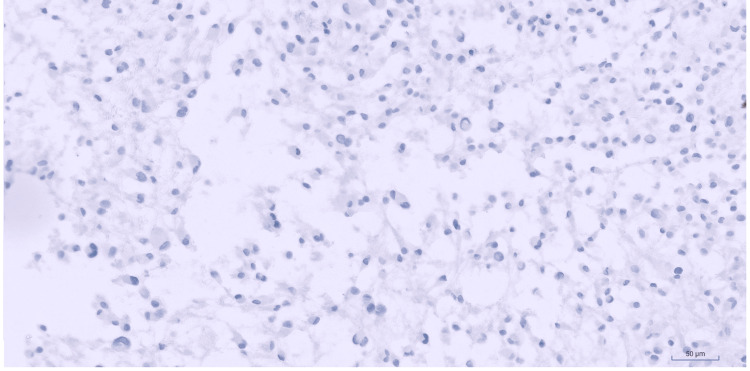
CD34 immunostain Negative CD34 immunostain (Immunohistochemistry (IHC), 20x).

Melanocytic markers such as S100, SOX10, HMB45, and MelanA, epithelial markers like Cytokeratin AE1/AE3 and CK 5/6, and other stains including CD34 (Figure 7), STAT6, ERG, P40, ALK1, MUC4, and WT1 are all negative. All the immunostains with their corresponding results are summarized in Table 1.

**Table 1:** Summary of immunostains with results.

Immunostains	Results
Desmin	Focal patchy positive
SMA (Smooth Muscle Actin)	Negative
SMMS (Smooth Muscle Myosin Heavy Chain)	Negative
H-Caldesmon	Negative
Myogenin	Negative
S100	Negative
SOX10	Negative
HMB45	Negative
MelanA	Negative
Cytokeratin AE1/AE3	Negative
CK 5/6 (Cytokeratin 5/6)	Negative
CD34	Negative
STAT6	Negative
ERG	Negative
P40	Negative
ALK1 (Anaplastic Lymphoma Kinase-1)	Negative
MUC4	Negative
WT1	Negative

Fluorescence in situ hybridization (FISH) for MDM2 gene amplification was negative. RNA fusion panel, a probe hybridization based next generation sequencing (NGS) assay that follows reverse transcription of extracted RNA with an approximate sensitivity of 1500 fusion transcripts, was negative for reportable or pathogenic gene fusions. Comprehensive tumor panel, a DNA sequencing assay that covers mutations in 547 genes using Illumina sequencing platform (NextSeq or NovoSeq), detected TRAF7 p.R641C gene mutation (c.1921C>T chr16:2226308:C:T 22.8% Substitution - Missense Tier 2), with no targetable variants, low mutation burden, and limited copy number alterations with 1-copy loss of CDKN2A/B and MTAP among minimal segmental chromosome/gains/losses (Table 2).

**Table 2:** Summary of molecular tests performed on the tumor FISH: Fluorescence in situ hybridization

Molecular Test	Results	Interpretation
FISH for MDM2 gene amplification	MDM2/CEP12 ratio < 2	Negative
RNA fusion panel	No reportable or pathogenic gene fusions	Negative
Comprehensive tumor panel	TRAF7 p.R641C gene mutation (c.1921C>T chr16:2226308:C:T 22.8% Substitution - Missense Tier 2), 1-copy loss of CDKN2A/B and MTAP via NGS CNV calling, Mutation burden: 1 (<25% percentile)	Pathogenic TRAF7 gene mutation, Focal monoallelic loss of CDKN2A/B and MTAP genes indicating limited copy number alterations, Low mutation burden

**Figure 8:**
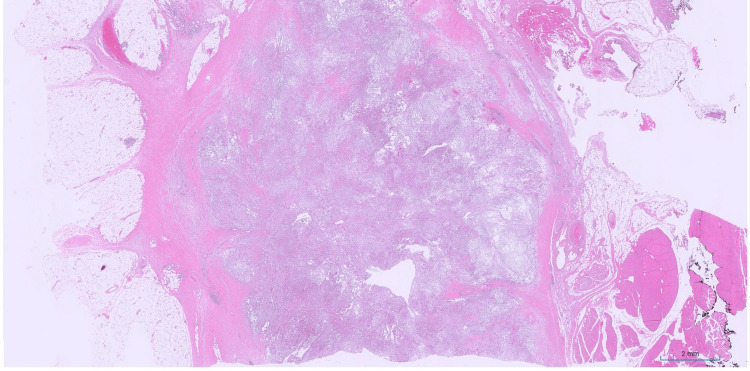
TRAF7-mutated myxoid mesenchymal tumor with epithelioid features - resection Low power magnification of the resection specimen demonstrating the relatively well circumscribed vaguely lobular nature of the tumor with peripheral lymphoid aggregates (hematoxylin and eosin (H&E), 0.5x).

Based on these findings, a TRAF7-mutated myxoid mesenchymal tumor with epithelioid features was rendered. Subsequently, the patient underwent complete wide local excision of the tumor with negative margins (closest margin was the deep soft tissue margin with 5 mm clearance) and no perioperative complications. A whole-body nuclear positron emission tomography (PET) scan was performed three weeks prior to surgery and demonstrated a markedly hypermetabolic focus compatible with the biopsy-proven neoplasm with a maximum standardized uptake value (SUV) of 9.5, and no suspicious tracer uptake elsewhere to suggest metastatic disease. Microscopic examination of the resection specimen revealed a relatively well circumscribed vaguely lobular cellular proliferation with peripheral lymphoid aggregates (Figure 8) and similar morphologic findings to the patient's prior biopsy.

**Figure 9:**
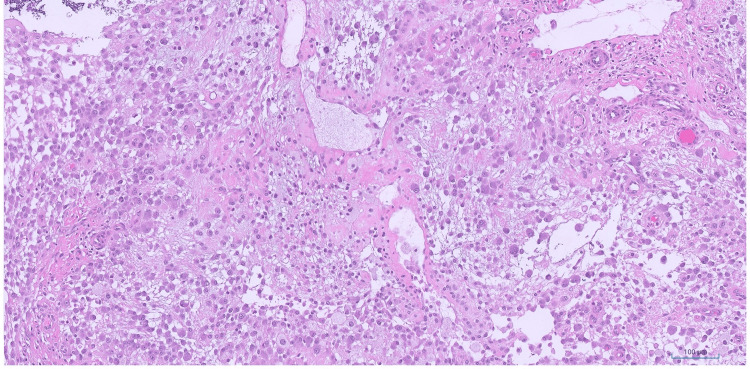
TRAF7-mutated myxoid mesenchymal tumor with epithelioid features - resection The majority of the tumor showed this epithelioid morphology set in a myxoid stroma with occasional perivascular hyalinization (hematoxylin and eosin (H&E), 10x).

**Figure 10:**
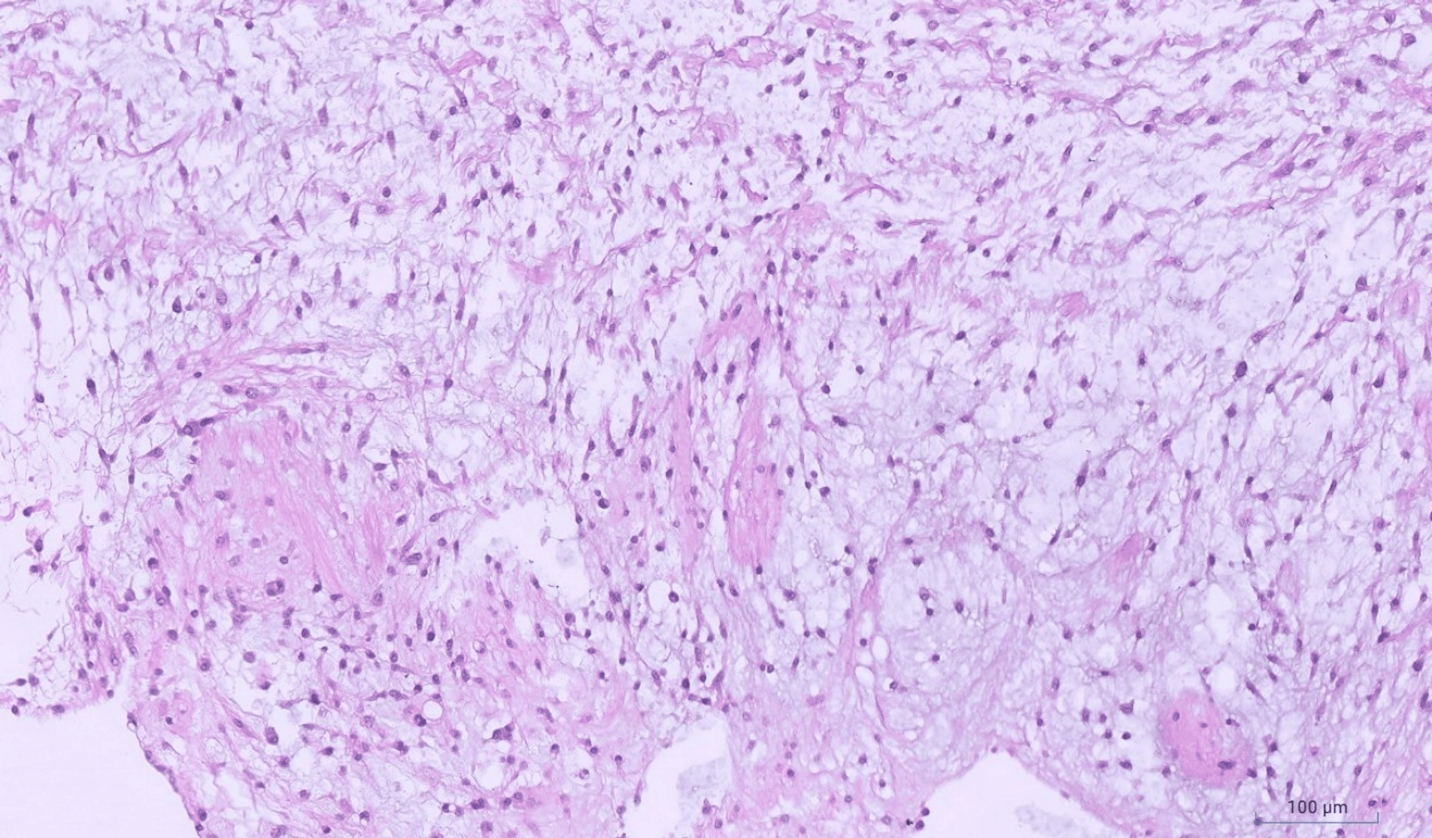
TRAF7-mutated myxoid mesenchymal tumor with epithelioid features - resection Very focal spindle cell areas with bland low-grade morphology were seen on the resection specimen (hematoxylin and eosin (H&E), 10x).

The tumor had predominantly (more than 95%) epithelioid morphology (Figure 9) with very focal spindle cell features (Figure 10) estimated at less than 5% of the tumor.

Mitotic figures reached up to two per 10 high-power fields and there was no evidence of necrosis. All margins were negative, and clinically, the decision was to closely follow up the patient given the uncertain biologic potential of this novel and emerging entity. Currently, at one-month post-resection, the patient is healing relatively well with a plan to see the patient back at three months post-op with repeat imaging and scans, after which the patient will be followed up closely clinically at six-month intervals.

## Discussion

*TRAF7* gene resides on chromosome 16p13.3, contains 21 exons, and is involved in ubiquitination by mediating the mitogen-activated protein kinase (MAPK) and nuclear factor kappa-light-chain-enhancer of activated B cells (NFKB) signaling pathways [1,5]. The WD40 repeats of *TRAF7* bind *MEKK3*, which in turn phosphorylates the *TRAF7* N-terminal region and induces ubiquitination [5]. TRAF7, in conjunction with *MEKK3,* results in a synergistic activation of NFKB and AP1, which impinge on the activation of *JNK* and p38 MAP kinases via intermediate effectors [5].

*TRAF7* mutations are associated with cardiac, facial, and digital anomalies with a developmental delay [5]. Interestingly, a de novo *TRAF7* mutation was also identified in a patient with autism [6]. Additionally, *TRAF7* somatic mutations have been reported in different types of neoplasms including intraneural perineuriomas, adenomatoid tumors, mesotheliomas, and meningiomas [2,7-10].

Literature review

Recently, a rare *TRAF7*-mutated myxoid spindle cell tumor was reported [3]. This initial report identified three cases with an aggressive clinical course involving the deep soft tissue in adult patients with median age of 67 years [3]. The tumors were infiltrative, involved the shoulder, chest wall and thigh and measured between 7 cm and 9.1 cm in maximum dimension. All three tumors showed via DNA sequencing *TRAF7* mutation as the sole somatic mutation with focal or limited copy number alterations, and no evidence of pathogenic gene fusions by RNA fusion panel. The tumors were reported to have a similar low-grade uniform spindle cell morphology without evidence of mitosis or necrosis set in fibrous and myxoid stroma with one of the tumors harboring higher grade epithelioid morphology with increased mitotic activity in the pleural metastatic focus only. The tumors showed focal non-specific staining for SMA or CD34 immunostains, and clustered on methylation profiling with myxofibrosarcoma and undifferentiated sarcoma methylation classes.

Interestingly, a recent report of a pediatric *TRAF7*-mutated myxoid and spindle cell mesenchymal tumor was described [4]. The tumor measured 12.6 cm, originated from the bowel wall, and similar to the initially reported adult cases had low-grade uniform spindle cell morphology without evidence of mitotic activity or necrosis within a myxoid matrix with an adjacent hypercellular area showing significant cytologic atypia and increased mitotic activity reaching up to 13 mitoses per 10 high power fields without evidence of necrosis. The authors described this hypercellular area to closely resemble myxofibrosarcoma. Whole exome sequencing of the tumor detected a somatic *TRAF7* gene mutation, while targeted next-generation sequencing and microarray analysis demonstrated 1q gain, 5p loss, 9p gain, 13q loss and monoallelic loss of chromosome 17 [4]. RNA fusion panel was negative for pathogenic gene fusions. The tumor cells were only positive for CD34 immunostain, and although methylation profiling did not match with any known methylation classes, the tumor clustered near undifferentiated pleomorphic sarcoma, myxofibrosarcoma, and leiomyosarcoma classes via unsupervised clustering [4]. Despite this initial presentation, the patient remained stable seven months after the mass resection and the tumor is speculated to potentially have an indolent clinical course.

Morphologic and molecular case comparison

When comparing the above reported *TRAF7*-mutated mesenchymal tumors to our case, we note the close overlap in the molecular profile including *TRAF7 *gene mutation being the only pathogenic somatic alteration detected on our sequencing panel and the presence of limited copy number alterations. On the morphologic level our case showed predominant epithelioid morphology as the primary morphologic feature with very focal spindle cell areas seen and prominent myxoid change. Additionally, our case demonstrated perivascular condensation, which is a pattern commonly described in myxofibrosarcoma. These findings along with the tumor’s non-specific immunophenotype and absence of specific line of differentiation raised the possibility of epithelioid variant of myxofibrosarcoma as a diagnostic consideration. Not surprisingly, myxoid sarcomas including myxofibrosarcoma and low-grade fibromyxoid sarcoma were described as close morphologic mimics in the reported *TRAF7*-mutated myxoid and spindle cell mesenchymal tumors [3-4]. However, despite the tumor morphology being reminiscent of epithelioid variant of myxofibrosarcoma in our case; the absence of reported *TRAF7* mutations in myxofibrosarcomas essentially excluded such consideration [11].

Our case, in addition to its striking epithelioid morphology, does deviate from the prior reported *TRAF7*-mutated myxoid and spindle mesenchymal tumors by being relatively small (2.8 cm in maximum size) and demonstrating patchy non-specific desmin staining with negative staining of all myoid, myoepithelial, melanocytic and epithelial markers. However, the immunostaining pattern was non-specific in all reported *TRAF7*-mutated myxoid and spindle mesenchymal tumors cases thus far with variable staining for SMA or CD34. In or case, the tumor showed similar non-specific staining for desmin. In the initial report by Dermawan et al. [3], all three reported cases had a spindle cell morphology unlike the epithelioid morphology in our case, however, one case reportedly harbored an epithelioid morphology in the metastatic focus thus making our case the first primary *TRAF7*-mutated myxoid mesenchymal tumor to harbor predominant epithelioid features as a primary presentation. Although methylation profiling is not available at our institution, we speculate our tumor will have similar methylation profile class characteristics either within or near myxofibrosarcoma, undifferentiated sarcoma, and leiomyosarcoma methylation classes.

Differential diagnosis

Other differential diagnosis to consider based on tumor morphology and patchy desmin staining include diffuse type tenosynovial giant cell tumor; however, the negative RNA fusion panel for the characteristic *CSF1* gene fusion commonly described in tenosynovial giant cell tumors and the novel *TRAF7* mutation argued against such differential. Myxoinflammatory fibroblastic sarcoma also enters the differential diagnosis; however, the predominant epithelioid morphology, the presence of only focal scattered inflammatory cells and the absence of reported TRAF7 mutations in myxoinflammatory fibroblastic sarcomas essentially excluded it.

## Conclusions

In conclusion, we present the first documented case in the published literature of primary *TRAF7*-mutated myxoid mesenchymal tumor with predominant epithelioid morphology, thus expanding the morphologic spectrum of *TRAF7*-mutated myxoid mesenchymal tumors. Given the low number of reported cases and its variable clinical course, the biologic potential of *TRAF7*-mutated myxoid mesenchymal tumors is regarded as uncertain at this point in time.
